# Leaderless secretory proteins of the neurodegenerative diseases via TNTs: a structure-function perspective

**DOI:** 10.3389/fnmol.2023.983108

**Published:** 2023-06-15

**Authors:** Sreedevi Padmanabhan, Ravi Manjithaya

**Affiliations:** Autophagy Laboratory, MBGU, JNCASR, Bangalore, India

**Keywords:** autophagy, neurodegenerative disease, leaderless proteins, intrinsic disordered regions, tunneling nanotubes, unconventional protein secretion

## Abstract

Neurodegenerative disease-causing proteins such as alpha-synuclein, tau, and huntingtin are known to traverse across cells via exosomes, extracellular vesicles and tunneling nanotubes (TNTs). There seems to be good synergy between exosomes and TNTs in intercellular communication. Interestingly, many of the known major neurodegenerative proteins/proteolytic products are leaderless and are also reported to be secreted out of the cell via unconventional protein secretion. Such classes contain intrinsically disordered proteins and regions (IDRs) within them. The dynamic behavior of these proteins is due to their heterogenic conformations that is exhibited owing to various factors that occur inside the cells. The amino acid sequence along with the chemical modifications has implications on the functional roles of IDRs inside the cells. Proteins that form aggregates resulting in neurodegeneration become resistant to degradation by the processes of autophagy and proteasome system thus leading to Tunneling nanotubes, TNT formation. The proteins that traverse across TNTs may or may not be dependent on the autophagy machinery. It is not yet clear whether the conformation of the protein plays a crucial role in its transport from one cell to another without getting degraded. Although there is some experimental data, there are many grey areas which need to be revisited. This review provides a different perspective on the structural and functional aspects of these leaderless proteins that get secreted outside the cell. In this review, attention has been focused on the characteristic features that lead to aggregation of leaderless secretory proteins (from structural-functional aspect) with special emphasis on TNTs.

## Introduction

Secretion is a pivotal physiological process that helps in the homeostasis of cells. Proteins synthesized inside the cells get secreted to the exterior by traversing across multiple organelle compartments which is directed by a small peptide tag called the leader peptide or the signal peptide. Multiple molecular machinery is involved in the transport of these proteins that are secreted out by passing through the ER, Golgi and plasma membrane and the process is termed as conventional protein secretion (CPS). As exclusions always occur, there are a subset of proteins that get secreted outside the cells without the presence of the leader peptide and this process is termed as unconventional protein secretion (UCPS). Unconventional protein secretion follows majorly four different pathways – Type I, Type II, Type III, and Type IV (Rabouille et al., 2012; Rabouille, 2016). Of these, type I and type II are non-vesicular. The vesicular pathways are mediated by the Type III and IV systems. Besides these modes of secretion, there exists another mode via tunneling nanotubes (TNTs) by which the proteins get transported from one cell to the other. Biological molecules including lipid vesicles, proteins, DNA and even organelles get transported through these TNTs.

It is interesting to note that the secreted proteins are often found to evolve faster than the intracellular proteins in mammalian cells (Julenius and Pedersen, 2006; Liao et al., 2010; Homma et al., 2018). Proteins that lack stable tertiary structure in their native form are termed as intrinsically disordered proteins and regions (IDRs) and they constitute a major fraction of the eukaryotic proteome (Dunker et al., 2013; Walsh et al., 2015). Intrinsic disorder of proteins results in structural diversity which can lead to multiple functions (multispecificity) which includes protein promiscuity and moonlighting functions and various pathologies (Hult and Berglund, 2007; Gupta et al., 2019; Blundell et al., 2020). Hence, the disorder becomes an intrinsic function since their presence is seen at significant levels in proteins involved in various regulatory processes such as signal transduction, transcription, DNA repair and chromatin remodeling (Blundell et al., 2020). Around 33% disorder is observed in the eukaryotic proteome, of which approximately 67% constitutes the long IDRs in the protein which are generally 30 or more amino acids long. They evolve faster than structured domains (Oldfield and Dunker, 2014). As the IDRs have more hydrophilic properties than structured regions, they are expected to be soluble in aqueous solutions (Uversky et al., 2000; Oldfield et al., 2005; Haynes et al., 2006; Uemura et al., 2018). However, *in silico* predictions employed to discriminate a short IDR (<11 residues, below the lower quartile point) from the long IDRs (>77 residues above the higher quartile point) demonstrated that the high disorder group was biased towards the lower solubility fraction while the low disorder group towards higher solubility fraction (Uemura et al., 2018). Recent studies by Pritisanac and colleagues suggest that the information in IDR sequences cannot be fully revealed only by positional conservations at the sequence level but conformational entropy can be modulated to facilitate IDRs to tune their energy landscapes thereby enabling diverse functional interactions and modes of regulation (Pritišanac et al., 2019). As IDRs play important roles in signaling processes (Forman-Kay and Mittag, 2013; Chong and Forman-Kay, 2016) and have implications in disease conditions (Babu, 2016; Chong and Forman-Kay, 2016; Alberti and Carra, 2018; Forman-Kay et al., 2018), we propose plausible role of the IDRs in the formation of TNTs in this review.

### Intercellular communication—role of extracellular vesicles, exosomes and TNTs in unconventional protein secretion

Eukaryotic cells employ several means of intercellular communication to address the changing physiological demands/cues and also in protecting cells from debilitating diseases and /or from pathogenic invasions. Intercellular communication is mediated by cell derived nano extracellular vesicles such as exosomes in a paracrine fashion and also by long range intercellular cytoplasmic bridges such as TNTs. Exosome proteins include the transmembrane proteins (CD9, CD63, CD81, CD82), heat shock proteins (HSC70, HSP60, Hsp70, Hsp90), proteins involved in MVB processing (Alix, TSG101), cytoskeleton proteins (actin, tubulin, cofilin, profilin, fibronectin, etc.), fusion/transport proteins (Annexins, Rabs), integrins, signal transduction proteins, immune regulatory molecules (MHC I and II) and various metabolic enzymes. Exosomes are lipid bilayer vesicles with a diameter of 30–150 nm, which can carry specific proteins, lipids, mRNA, miRNA and other substances. The early endosomes mature into late endosomes and multivesicular bodies (MVBs), gets translocated to plasma membrane and secrete out as exosomes (Colombo et al., 2014). Tunneling nanotubes (TNTs), discovered in 2004, are thin, long protrusions between cells utilized for intercellular transfer and communication. These newly discovered structures have not only been demonstrated to play a crucial role in homeostasis, but also in the spreading of diseases, infections, and metastases. TNTs are actin-based transient cytoplasmic extensions which are stretched between cells in the form of open ended nanotubular channels (50–200 nm; Rustom et al., 2004) that transport cargoes across cells. Motor proteins play as a common component in both TNTs and EVs such as exosomes in the transport of cargoes. Exosomes and TNTs resemble the pattern in disseminating the disease associated proteins especially in the neurodegenerative disease pathologies. The role of TNTs and EVs are implicated in the spread of misfolded protein aggregates between different cells in the central nervous system. The role of EVs in intercellular communication is relatively well-understood but the role of TNTs is largely underexplored. The extracellular vesicles and TNTs are structurally different but perform the parallel process of intercellular communication. In the absence of direct physical contact, cells communicate with each other using long range TNTs or by secreting EVs or exosomes in a paracrine fashion. A comparative profile of the EVs and TNTs in intercellular communication w.r.t neurodegenerative diseases are listed in Table 1.

**Table 1:** Comparison between the EVs and TNTs in intercellular communication in neurodegeneration.

Characteristics	EVs, exosomes	TNTs
Structure/form of protein transport	Proteins are transported in the vesicles.	Proteins are transported via nanotubes.
Cargo transport	EVs transport bioactive molecules, including nucleic acids, proteins, lipids, and metabolites.	TNTs transport cellular organelles such as mitochondria, lysosomes, vesicles, biomolecules such as proteins, lipid droplets, ions.
Composition	EVs and exosomes are cargo carriers which is composed of lipids and proteins.	TNTs are actin-based transient cytoplasmic extensions which are stretched between cells in the form of open ended nanotubular channels.
Size	The size is heterogenous which ranges from 30–10,000 nm.	The tubular channels have a size of 50–200 nm. Nanotubes less than 0.7 μm in diameter, mainly containing actin and nanotubes larger than 0.7 μm, containing both microfilaments of actin and microtubules are observed.
Mode of communication	Cell derived nanovesicles working in paracrine fashion.	Long range direct contact-based communication bridges/tubes.
Requirement of motor proteins	Actin and myosin proteins are involved in the transport of these nano vesicles.	TNTs are actin-based tubes through which the cargoes are transported with the help of motor proteins.
Role in disease propagation	Involved in the transport and propagation of disease-causing aggregates.	Involved in the transport and propagation of disease-causing aggregates.
Speed of transfer of aggregate proteins	Depends on the motor proteins.	Ranges between 0.1–15 μm/s.

### Factors associated with the TNT formation and transport of leaderless secretory proteins via TNTs

This review attempts to understand the common underpinnings that work in unison for the secretion of the leaderless peptides with special emphasis on TNTs that is largely unknown. In this regard, some of the factors which have the putative roles are discussed here along with the evidences from the known literature.

## Intrinsically disordered regions in leaderless secretory proteins

**Figure 1:**
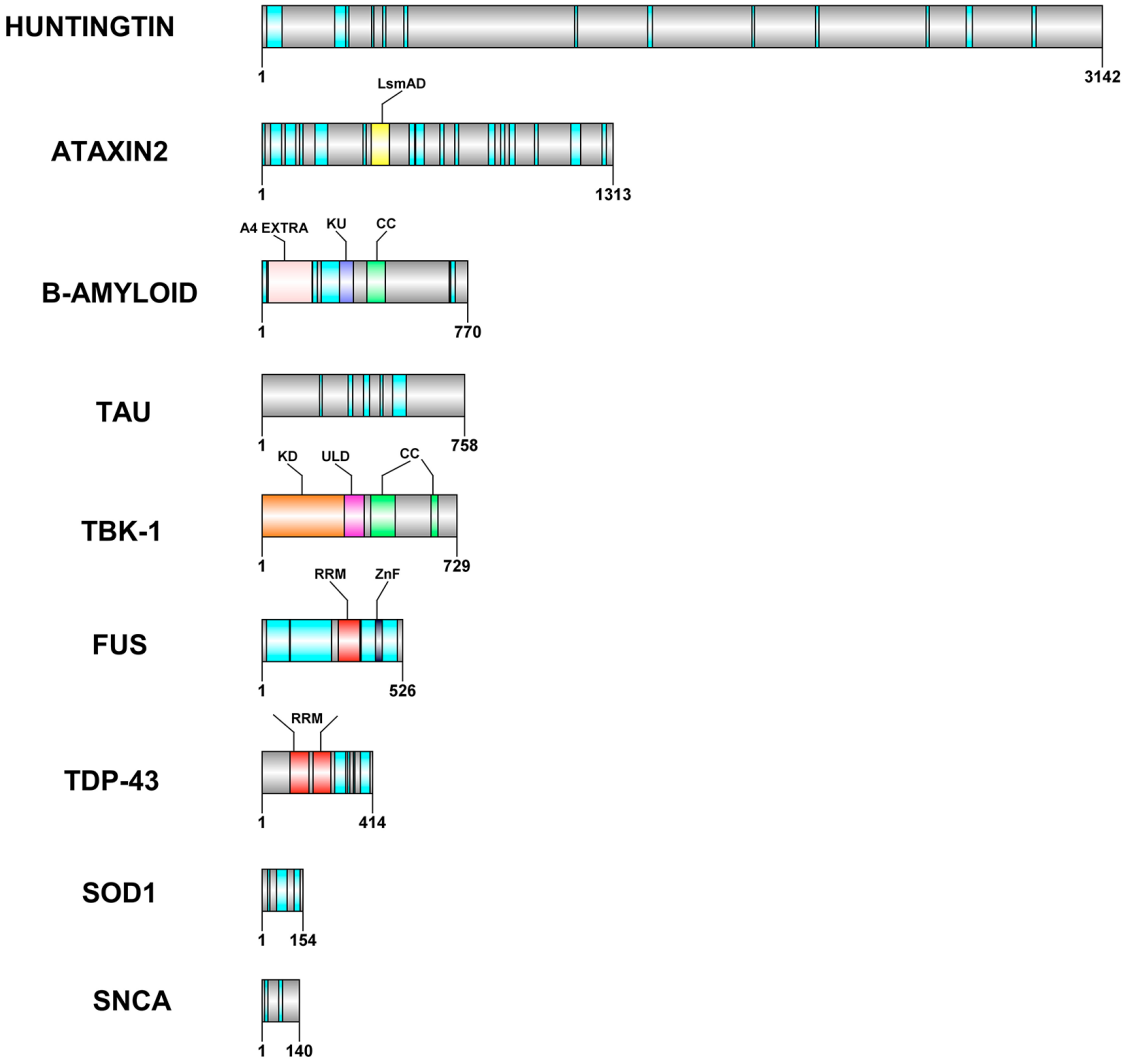
Domain architecture of some of the leaderless proteins that are involved in neurodegenerative diseases. RRM—RNA Recognition motif is colored in red, the IDRs in cyan, CC—coiled coil structures in light green color. ZnF−zinc finger motif is colored in grey color, LsmAD domain in yellow color interacts with RNA helicase, KD-Kinase domain in orange color, ULD—Ubiquitin-Like domain in dark pink color, A4 extra in light pink color and KU domain in dark blue color. The domain architecture was drawn to scale using DOG 2.0 illustrator (Ren et al., 2009).

Proteostasis is maintained by multiple cellular pathways such as ubiquitin-proteasome system (UPS), autophagy-lysosomal system and unfolded protein response (UPR) in neurons. All these pathways regulate processes such as protein folding, disaggregation, degradation and extracellular release of misfolded or disease related proteins (Lim and Yue, 2015). It is interesting to note that many of the proteins/proteolytic products associated with neurodegeneration are leaderless and are also reported to be secreted out of the cell via unconventional protein secretion. For example, fragmentation of specific regions such as the central region of Tau can be pathogenic (Xia et al., 2021) and C-truncated α-syn is found to be associated with toxicity (Sorrentino and Giasson, 2020). Several lines of evidence implicate the secretion of these proteins, that form aggregates, in disease progression. The disorder and promiscuity of these proteins seems to affect both communicable and non-communicable diseases (Blundell et al., 2020). Several neurological diseases (conformational diseases) are a result of the key factors of IDRs due to their higher propensity of aggregation (Mukherjee and Gupta, 2017). The IDR mediated aggregation leads to disease impacting amyloid formation (Uversky et al., 2000, 2008; Hammerstrom, 2009; Sweeney et al., 2017). Proteins such as huntingtin, TDP-43, FUS, SOD1, ataxin-2, alpha synuclein, beta amyloid, and tau contain intrinsically disordered regions (IDRs) within them (Table 2; Figure 1) which are one of the pivotal common factors causing their aggregation. The dynamic behavior of these proteins is due to their heterogenic conformations that is exhibited due to various factors that occur inside the cells. The amino acid sequence along with the chemical modifications has implications on the functional roles of IDRs inside the cells. In general, absence of hydrophobic residues, but presence of regions of high fraction of charged residues and aromatic amino acids are causes of disorder in intrinsically disordered proteins (Oldfield and Dunker, 2014; van der Lee et al., 2014; Uversky, 2019). It is observed that many of the proteins involved in the calcium signaling pathways (e.g., the N-methyl-D-aspartate, NMDA receptors in spine) are intrinsically disordered. The intrinsically disordered C-terminal in the NMDA receptor regulates calcium signaling pathway and trafficking by altering the properties of the channel (Warnet et al., 2021). This long C-terminal IDR also serves as a scaffold to assemble the downstream signaling proteins, including calmodulin, kinases and calcineurin. This means that the role of IDRs in various intracellular proteins is pivotal in regulatory mechanisms that contribute to cellular signaling (Bondos et al., 2022).

**Table 2:** Comprehensive list of leaderless proteins involved in neurodegenerative diseases.

Neurodegenerative disease	Protein aggregates (Uniprot ID)	Length of the protein	IDR protein structure	Species localization	Autophagy secretion-presence of LIR motif*	Unconventional secretion	TNT mode secretion	References
Huntington’s disease	Huntingtin (P42858)	3,142	PolyQ	Intracellular (cytosolic and nuclear)	Yes	Yes	Yes	Sharma and Subramaniam (2019)
Amyotrophic lateral sclerosis	TDP-43(Q13148)	414	C-terminal domain	Cytoplasmic aggregates	Yes	Yes	Yes	Tardivel et al. (2016)
	FUS (P35637)	526	N-terminal domain		Yes	Not reported	Not reported	-
	SOD1 (P00441)	154	22–30,55–95 region; 121–143 region		Yes	Yes	Yes	Abounit et al. (2016)
	Ataxin-2(Q99700)	1,313	Poly Q-tract		Yes	Not reported	Yes	-
	TBK-1 (Q9UHD2)	729	TBK-1		Yes	Not reported	Involved in TNT secretion	-
Parkinson’s disease	Alpha-synuclein (P37840)	140	C-terminal domain	Intracellular LB formation, extracellular and membrane	No	Yes	Yes	Abounit et al. (2016)
Alzheimer’s disease	Beta-amyloid (P05067)	770	Beta-amyloid	Extracellular plaques	Yes	Yes	Yes	Dilna et al. (2021)
	Tau (P10636)	758	N-terminal domain	Intracellular neurofibrillary tangles	Yes	Yes	Yes	Tardivel et al. (2016)

* https://ilir.warwick.ac.uk/lirpredict.php and data from Padmanabhan et al. (2018).

In the past few years, the prevalence and roles of intrinsically disordered proteins and IDRs in synaptic vesicle trafficking and exocytosis in addition to synaptic organization has gained more attention (Snead and Eliezer, 2019). Although IDPs lack stable tertiary structures, they retain biological function and activity of the proteins such as cell signaling. Although the significance of IDRs is well-established in other areas of biology, it is not well-explored in the field of neurobiology. Similar lines of evidence propose the role of intrinsically disordered proteins in dictating and modulating the cellular phenotype depending on the environmental cues/micro environment in the intercellular communication (Kulkarni et al., 2022). It is due to the above reasons, there is a proposed role of IDRs in information transfer as linkers, effectors, and sensors that enable complex regulatory behavior in molecular signaling (Hilser and Thompson, 2007; Motlagh and Hilser, 2012; Tompa, 2014; Arbesú et al., 2018; Follis et al., 2018; Li and Hilser, 2018; Zhang et al., 2018). Hence, it is important to understand as to whether the IDRs in the leaderless proteins have any role in the TNT formation especially in the neurodegenerative diseases. It is not known as to whether the IDRs are dictating the TNT formation.

## Charged residues

The proportion of charged residues are high in the primary sequences of IDPs with few hydrophobic amino acids (Cortese et al., 2008; Uversky, 2016). It is the unique properties of the IDR that render conformational and functional flexibility. The structural complexity is brought about by these simple sequences and their inability to spontaneously fold into a unique three-dimensional structure. The charge content, their pattern within the IDRs along with the sequence composition are the determining factors that dictate the IDPs to respond to external factors like ionic strength and temperature (Shammas et al., 2016). The IDPs demonstrate flat, free energy landscapes with local minima separated by low barriers, and they tend to rapidly oscillate between different disordered conformations (Papoian, 2008). Post translational modifications alter the energy landscape and the resultant conformational ensemble of the IDP, and they modulate interactions with other biomolecules (Bah and Forman-Kay, 2016; Guharoy et al., 2016; Shammas et al., 2016; Uversky, 2016). Recent report from Ferreira et al. (2022) have demonstrated that pentapeptide KFERQ containing proteins are loaded onto a subpopulation of exosomes. This process which is ESCRT independent is dependent on HSC70, CD63, Alix, syntenin-1, Rab31 and ceramides. On a similar note, the environmental conditions such as hypoxic conditions induce TNTs in glioblastoma and surrounding non-tumor astrocytes (Valdebenito et al., 2021). It is important to note that as the evolutionary signatures in the amino acid sequences dictate the molecular features (Zarin et al., 2019) suggesting charged residues / net charge of the proteins might have some role in TNTs.

## Prion-like proteins

The prion-like proteins such as huntingtin fibrils and TDP-43 involved in neurodegenerative diseases are shown to trigger TNT formation in neuronal cells (Gousset et al., 2009; Costanzo et al., 2013; Ding et al., 2015). Human prion-like proteins often correspond to nucleic acid binding proteins, displaying both globular domains and long intrinsically disordered regions (IDRs; Harrison and Shorter, 2017). Their IDRs are of low complexity and are usually enriched in Gln and Asn residues and depleted in hydrophobic and charged residues and these sequence stretches are called prion-like domains (PrLDs). These domains help the prion-like proteins aggregate into amyloid fibrils, which then can accommodate incoming protein monomers as seeds, thus propagating the polymeric fold. Human prion-like proteins are gaining wide attention as they are identified in an increasing number of neurodegenerative diseases in the form of insoluble inclusions (Harrison and Shorter, 2017). Some of the well-characterized examples are FUS, TDP-43, TAF15, EWSR1, TIA1, hnRNPA1, and hnRNPA2 proteins. All the neurodegenerative disorders such as Alzheimer’s disease (AD), Parkinson’s disease (PD), amyotrophic lateral sclerosis (ALS), or transmissible spongiform encephalopathies (TSEs) are the result of these self-propagating prion-like properties that disrupt cellular proteostasis (Jucker and Walker, 2013; Liberski, 2014; Goedert, 2015). Understanding the exact mechanisms of cell-to-cell spreading of pathological species are still a subject of intense research. The role of TNTs in such propagation has been demonstrated in Huntington’s disease, PD and ALS/fronto-temporal dementia among other known mechanisms (Aguzzi and Lakkaraju, 2016). The amyloid Aβ peptide has been shown to traffic through TNTs and induce cytotoxicity in AD (Wang et al., 2011). Similarly, recent reports demonstrate that the intercellular spread of pathological tau is facilitated by the increased formation of TNTs by the extracellular tau (Tardivel et al., 2016) which suggests the plausible role of these proteins in TNT formation.

## RNA-binding property

It is interesting to note that many of the RNA-binding proteins found in stress granules contain aggregation-prone prion-like domains (PrLDs) which are rich in glutamine/asparagine. Several mutations in the PrLD-containing RNA-binding proteins have been implicated in various neurodegenerative diseases including ALS and FTD (Ramaswami et al., 2013; Baradaran-Heravi et al., 2020). Incidentally, several parallel reports suggest the secretion of many RNA-binding proteins (~204) that are microtubule-associated protein light chain 3 (LC3)-mediated, and are termed as LC3-Dependent EV Loading and Secretion (LDELS; Leidal et al., 2020; Leidal and Debnath, 2020). In a recent extended study of these data sets, Biswal et al. (2022) observed that of the 204 proteins, 202 of them were found to be leaderless and it has been suggested that the triacidic motif (EEE/DDD/DEE) is found to occur in a statistically significant proportion. The plausible role of phosphorylatable amino acid and the proximity of LC3-interacting region (LIR) in autophagy-dependent unconventional protein secretion has been reported. This statistically significant observation of the presence of triacidic motif in mammalian proteins is an extension/ supporting study of the diacidic motif—DE in UCPS (Cruz-Garcia et al., 2017) and the context in which the diacidic motif appears (Padmanabhan et al., 2018). Studies demonstrate that the RNA-binding protein, nucleolin interacts with the known TNT-inducing protein, MSec and this interaction forms the basis for TNT formation in mammalian cells. This is brought about by the RNA-binding domains (RBDs) of nucleolin, which in turn maintains the cytosolic levels of 14–3-3ζ mRNA, thus helping in TNT formation (Dagar et al., 2021). This suggests a plausible nexus between LC3-dependent secretion, RNA-binding proteins and TNTs.

## Role of actin and myosin proteins in TNTs

Proteins that form aggregates and cause neurodegeneration become resistant to degradation by the processes of autophagy and proteasome system thus leading to TNT formation. The proteins that traverse across TNTs may or may not be dependent on the autophagy machinery. It is not yet clear whether the conformation of the protein plays a crucial role in its transport from one cell to another without getting degraded. Starvation induced autophagy has been shown to increase the formation of TNTs in mesothelioma cells (Lou et al., 2012), suggesting a correlation between these processes. Recently, it was observed that TNT signaling induces the secretion of pro-survival cytokines (Polak et al., 2015). TNT formation is also dependent on actin (Rustom et al., 2004; Polak et al., 2015) and this co-dependence might be important for autophagosome transport through the TNT machinery. TNTs are membranous protrusions supported by filamentous actin that mediate continuity between remote cells by remaining open at both ends for cargo transport. Since actin is involved in all the steps of autophagy (Aplin et al., 1992; Reggiori et al., 2005; Aguilera et al., 2012; Zhuo et al., 2013; Kast and Dominguez, 2017), there could be a plausible crosstalk between autophagy and TNTs. Amyloid-β-induced membrane damage triggers TNTs by exploiting p21-activated kinase-dependent actin remodulation (Dilna et al., 2021). It is demonstrated that actin related proteins have opposite effect on TNT formation in differing cell types such as immune cells. Some of the actin and membrane related proteins that effects TNTs are IRSp53, CDC42, Rac1, VASP, Fascin, Eps8 and My010 (Ljubojevic et al., 2021). The insulin receptor tyrosine kinase substrate of 53 KDa (IRSP53) is an I-BAR protein that is involved in the initiation and stabilization of negative membrane curvature. Cell division cycle 42, CDC42 is a Rho-GTPase signaling protein that is involved in triggering actin rearrangement in TNTs. The Ras-related C3 botulinum toxin substrate 1 (Rac1) is involved in actin related protein, Arp2/3 activation. The vasodilator-stimulated phosphor protein (VASP) is an actin nucleator that elongates straight actin filaments. The epidermal growth receptor substrate 8 (Eps8) has a dual role in limiting actin protrusion extension and in stabilization of TNTs. Eps8 acts as a positive regulator of TNT formation. Fascin is an actin filament bundling protein. The unconventional myosin, Myo10 is found to be a key regulator of TNT formation in neuronal cells (Gousset et al., 2013). All the above-mentioned molecular players seem to play in concert with each other along with the 14-3-3 protein in the TNT formation (Ljubojevic et al., 2021). As 14-3-3 is also one of the key modulators of autophagy, there is a plausible interlink between the process of autophagy and TNT formation.

## Role of GTPases in TNTs

The role of Rho-GTPases is implicated in cell surface dynamics and TNT biogenesis (Raghavan et al., 2021) and is observed to be significant in various physiology and disease (Zhang et al., 2020). Although TNT formation in neurons is not completely understood, studies have illustrated that the mutant huntingtin could be transported through the biogenesis of TNTs mediated by the brain-enriched GTPase/SUMO E3-like protein Rhes (Sharma and Subramaniam, 2019). The involvement of small Rho GTPases like Cdc42, needs to be established in the context of cell-to-cell transmission of prion and prion-like proteins. The complex relationship between Rho GTPases and actin-regulatory complexes in neurons can be brought about by the plausible fact that Cdc42-mediated pathway via IRSp53 and VASP inhibits TNT formation by promoting the extension of filopodia concomitantly (Delage et al., 2016). The potential causal associations with neurodegenerative disease development and progression needs to be determined despite the established transfer of pathological agents in neuronal models. The interaction of M-Sec with RalA GTPase is necessary for the formation of TNTs (Hase et al., 2009). As the exocyst complex is involved in vesicle trafficking and is involved in autophagy process and secretion (Singh et al., 2019; Krause et al., 2022), it is plausible that the directed delivery of membrane to a growing TNT is needed; however, the exact mechanism is not well known (Ljubojevic et al., 2021).

## IDRs of GRASPs and TNTs

Golgi reassembly stacking proteins (GRASP 55 and 65) are known to regulate unconventional protein secretion. Golgi apparatus is known to undergo fragmentation by the phosphorylation of GRASP 55 and 65 during cell division and are known to play pivotal regulatory roles in UCPS. GRASP55 regulates the unconventional secretion of mutant Htt (Ahat et al., 2022). The biophysical characterization of human GRASP65 proteins demonstrated its higher intrinsic disorder which is capable of forming temperature dependent amorphous aggregates as well as time-dependent amyloid fibrils (Reddy et al., 2020) which suggests that GRASPs might have a putative role in the aggregation of the proteins involved in neurodegenerative diseases. It would be interesting to check the involvement of GRASP proteins in TNT formation that requires experimental validation.

## TNTs and autophagy crosstalk—plausible pathways

There are several pathways that are involved in the secretion of neurodegenerative disease-causing aggregates. One such key cellular homeostatic machinery, autophagy, is pivotal and acts as a double-edged sword in neurodegenerative diseases (Shintani and Klionsky, 2004; Martinet et al., 2009). Autophagy plays a central role in the removal of protein aggregates within neurons as seen in diseases such as AD, Huntington’s, and PD (Rubinsztein et al., 2012). Autophagy-dependent secretion of neurodegenerative disease-causing aggregates such as α-synuclein, β-amyloid and tau that are leaderless proteins seem to have significant implications in disease pathology. It is known that autophagy plays a role in the secretion of β-amyloid aggregates formed in AD. Conditional knockdown of Atg7 in excitatory neuronal cells in mice was found to influence β-amyloid secretion, thereby impacting plaque formation, a pathological hallmark of AD (Nilsson et al., 2013; Nilsson and Saido, 2014; Nilsson et al., 2015). Although there are many common factors involved in the progression of disease via TNTs, there are no concrete studies that establish the direct link between autophagy and TNTs. On a similar note, the molecular machinery involved in membrane protrusion and stabilization of filopodia might have some common players in the TNT formation which needs experimental validation. The secretory machinery involving exocyst complex in membrane delivery to the TNTs and the regulation by 14-3-3 protein provides clues in the crosstalk of autophagy with TNT formation. The plausible role of this signaling molecule in TNT formation based on the local environmental cues needs experimental validation.

## Conclusion

Although many studies point towards the targeted proteolytic products of the precursor proteins and their involvement in neurodegenerative diseases, a significant limitation is the lack of truncation-specific monoclonal antibodies that can detect these fragments in the disease (Xia et al., 2021). Activation of various proteases that elevates the proteolytic products in blood and CSF can be harnessed as cardinal biomarker tools to understand the disease pathology, diagnosis, cellular pathways and mechanisms. Currently, as the experimental studies are limited, many grey areas still remain that need to be revisited to understand the crosstalk of autophagy, UCPS and TNTs w.r.t neurodegenerative diseases. This review provides a different perspective on the structural and functional aspects of these leaderless proteins that get secreted outside the cell via TNTs. TNTs have been proposed to play a key role in neurodegenerative diseases and are involved in the spread of aggregated proteins such as tau, APP, and huntingtin by an intracellular pathway instead of a soluble-mediated mechanism (Gousset and Zurzolo, 2009; Abounit et al., 2016; Grudina et al., 2019; Mittal et al., 2019). Cells naturally create connections such as TNTs through which cell survival as well as disease progression occurs. This opens up several avenues for researchers to explore these pathways and thereby provide therapeutic solutions such as nanomedicines (Ottonelli et al., 2022).

## Open questions

1. Although there is a good nexus between the cell homeostatic machinery, autophagy with neurodegenerative diseases and unconventional protein secretion, crosstalk between autophagy and TNTs, is there any direct evidence to support the formation of TNTs with leaderless proteins?
2. Is any selective autophagy machinery involved in TNT formation supporting UCPS?
3. Is there any specificity of the RNA-binding proteins in triggering TNT formation based on environmental cues?
4. Is there any synergy between autophagy machinery and TNT biogenesis?
5. Is there any role of GRASPs in TNT formation and in turn the UCPS?

## Author contributions

SP: conceptualization, writing—original draft preparation, review, and editing. RM: conceptualization, supervision, review, and editing. All authors contributed to the article and approved the submitted version.

## Funding

This study was supported by JNCASR intramural funds, DST−Science and Engineering Research Board (SERB) grant (EMR/2015/001946) to RM and Department of Biotechnology grant, Department of Biotechnology grant in Life Science Research, Education and Training at JNCASR (BT/INF/22/SP27679/2018). The financial support from the DBT-RA program in Biotechnology and Life Sciences and DST-SERB NPDF to SP is gratefully acknowledged.

## Conflict of interest

The authors declare that the research was conducted in the absence of any commercial or financial relationships that could be construed as a potential conflict of interest.

## Publisher’s note

All claims expressed in this article are solely those of the authors and do not necessarily represent those of their affiliated organizations, or those of the publisher, the editors and the reviewers. Any product that may be evaluated in this article, or claim that may be made by its manufacturer, is not guaranteed or endorsed by the publisher.
